# ﻿A new species of *Bundoksia* Lucañas, 2021 with comments on its subfamilial placement, based on morphological and molecular data

**DOI:** 10.3897/zookeys.1085.72927

**Published:** 2022-02-08

**Authors:** Yong Li, Xinxing Luo, Jiawei Zhang, Zongqing Wang, Yanli Che

**Affiliations:** 1 College of Plant Protection, Southwest University, Beibei, Chongqing 400715, China Southwest University Beibei China

**Keywords:** Bayesian Inference, cockroaches, DNA barcodes, haplotype network, Maximum Likelihood

## Abstract

One new species of *Bundoksia* Lucañas, 2021 from China is described. We construct a haplotype network from 21 *COI* sequences to display the relationships amongst populations of *Bundoksialongissima***sp. nov.**, mainly from Hainan Island, Yunnan Province and Guangxi Province, China. For the first time, we provide the details of female genitalia in addition to the known external morphology and male genitalia of the genus. Six molecular markers (*12S*, *16S*, *18S*, *28S*, *COI* and *COII*) from a total of 38 samples, including three samples of *Bundoksialongissima***sp. nov.**, are used to reconstruct phylogenetic trees using Maximum Likelihood (ML) and Bayesian Inference (BI) to assess the phylogenetic affinities of *Bundoksia*. Photographs of the morphology and a key to the three *Bundoksia* species are also provided.

## ﻿Introduction

The genus *Bundoksia* Lucañas, 2021 was established with *Bundoksiarufocercata* (Shelford, 1911) as type species, based on its smooth pronotum, flattened tibiae, the meso- and metafemur sparsely armed with dissimilarly-sized spines. Up to now, the genus *Bundoksia* contained two species, *Bundoksiarufocercata* and *Bundoksiasibuyania*, both distributed in the Philippines. Lucañas (2021) mentioned that the genus *Bundoksia* can be distinguished from *Cartoblatta* Shelford, *Shelfordella* Adelung and *Deropeltis* Burmeister by the specialised first abdominal tergite. Moreover, the genus *Bundoksia* possesses some of the characteristics of the subfamilies Archiblattinae and Blattinae.

*COI* has been recommended as a useful DNA barcode to solve the sexual dimorphism existed in cockroach (Yang et al. 2019) and judge intraspecific variation or interspecific difference for cockroaches (Li et al. 2020) combined with other data. In addition, use of multi-gene combinations to infer phylogenetic trees has gradually become an available tool to confirm the taxonomic status of cockroach genus (Djernæs et al. 2015). With the discovery of the new species *Bundoksialongissima* sp. nov., based on morphological and molecular data (*COI*), four mitochondrial markers (*12S*, *16S*, *COI* and *COII*) and two nuclear markers (*18S, 28S*) were sequenced to explore the phylogenetic affinities of the genus *Bundoksia*.

## ﻿Materials and methods:

### ﻿Taxon sampling

**Figure 1.**
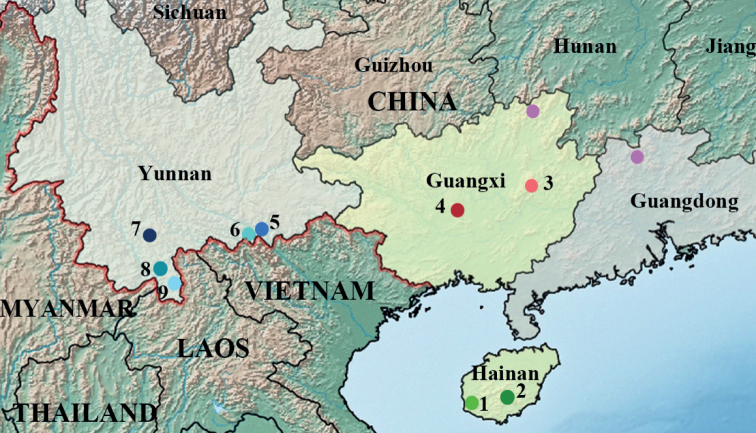
Geographic distribution of *Bundoksialongissima* sp. nov. Numbers for sampling localities match those in Suppl. material 1: Table S1. Different colours represent different populations. Purple circles indicate no molecular data.

Specimens were collected mainly from Yunnan, Hainan and Guangxi Province, China during 2014 to 2019 (Suppl. material 1: Table S1, Fig. 1). The samples were stored in absolute ethanol at −20 °C. All voucher specimens (Suppl. material 1: Table S1) were deposited in the Institute of Entomology, College of Plant Protection, Southwest University Chongqing, China (SWU). Voucher numbers and GenBank accession numbers are provided in Suppl. material 1: Table S1 and Suppl. material 2: Table S2.

### ﻿Morphological study

Morphological terminology used in this article mainly follows McKittrick (1964) and Li et al. (2013) for male and female genitalia, Roth (2003) and Li et al. (2018) for other characters. Terminology abbreviations in this study are as follow:

**ScP** subcostal posterior

**R** radius

**Cu** cubitus

**CuA** cubitus anterior

**CuP** cubitus posterior

**Pcu** postcubitus

**M** media

**V[1**], **V[s**] vannal veins

**L1**, **L2d**/**L2v**, **L3** sclerites of the left phallomere

**R1**, **R2**, **R3** sclerites of the right phallomere

**v.ph** ventral phallomere

**TX** tergum X

**pp.** paraprocts

**v.I.** first valve

**v.II.** second valve

**v.III.** third valve

**vlf.I** first valvifer

**p.l.** posterior lobes of valvifer II

**ltst.IX** laterosternite IX

**pt.** paratergites

**a.a.** anterior arch

**sp.pl.** spermathecal plate

**sp.o.** spermathecal opening

**sp.** spermatheca

**bsv.** basivalvula

**ltst.sh** laterosternal shelf

**vst.s** vestibular sclerite

**inst.f.** intersternal fold

Measurement data of the specimens were obtained by vernier caliper and Leica M205A microscopic system, such as body length including tegmen, body length, pronotum length × width, interantennal distance, interocular distance, head length × width, tegmina length, approximate length ratio of 3^rd^-5^th^ segments of maxillary palps. Genital segments of examined specimens were soaked with 10% sodium hydroxide (NaOH) for 10 minutes, observed in glycerol with a Motic K400 stereomicroscope and preserved with the remainder of the specimen in ethyl alcohol at −20 °C. The photographs of samples and genitalia were obtained by using a Leica M205A microscopic system. All of the images and photographs were processed in Adobe Photoshop CS6. Type materials are deposited in the Institute of Entomology, College of Plant Protection, Southwest University Chongqing, China (SWU).

### ﻿DNA extraction, amplification and sequencing

A total of 21 *COI* sequences of *Bundoksialongissima* sp. nov. were sequenced to determine the intraspecific variation, accession numbers: OM370873-OM370893 (Suppl. material 1: Table S1). The *COI* fragment was amplified by PCR, and PCR primers were as follows: *COI*-F3 (5’-CAACYAATCATAAAGANATTGGAAC-3’) and *COI*-R3 (5’-TAAACTTCTGGRTGACCAAARAATCA-3’). The conditions of amplification were: 98 °C initial denaturation for 2 min, followed by 35 cycles at 98 °C for 10 s, 51 °C for 10 s, and 72 °Cfor 10 s, with a final extension at 72 °C for 5 min. DNA was then sent to TsingKe Co. Ltd., Chongqing, China for sequencing.

We sequenced five additional markers of *Bundoksialongissima* sp. nov.: mitochondrial *12S*, *16S*, *COII* and nuclear *18S*, *28S* (Suppl. material 2: Table S2). We used an insect DNA extraction kit (D3121-02, Magen, Guangzhou, China) to extract the total DNA of examined specimens from hind-leg tissue. Total DNA was first stored at −20 °C then sent to TsingKe Co. Ltd., Chongqing, China for sequencing. The library generation and paired-end sequencing were completed on the Illumina HiSeq 2000 platform. Mitochondrial gene fragments *12S*, *16S*, *COII* and nuclear *18S* rRNA and *28S* rRNA were obtained by mapping sequence reads to the reference gene sequence of relative species, with the aid of Geneious Prime v.2021.1 (Biomatters Ltd., Auckland, New Zealand).

### ﻿Sequence alignment and phylogenetic analyses

A total of 27 *COI* sequences (excluding the primer, 658 bp), including 21 sequences of *Bundoksialongissimi* sp. nov. from Hainan, Yunnan and Guangxi in this study, along with others from GenBank corresponding to six outgroup species, were aligned using MEGA 7.0 (Kumar et al. 2007) and adjusted visually after translation into amino acid sequences. The genetic divergence values were calculated in MEGA 7 (Kumar et al. 2007) on the basis of the Kimura 2 - parameter (K2P) model (Kimura 1980). For Neighbour-Joining (NJ), implemented in MEGA 7 (Kumar et al. 2007), the outgroups contained six taxa (*Mantisreligiosa*, *Protagonistalugubris*, *Homalosilphaarcifera*, *Mimosilphadisticha*, *Homalosilphanigricans* and *Homalosilpha*sp.). In the meantime, *COI* data of *Bundoksialongissimi* sp. nov. were used to construct the haplotype network for inferring relationships amongst different populations, which were constructed in the software PopART v.1.7 (Leigh and Bryant 2015).

The rest of the five markers (*12S*, *16S*, *COII*, *18S* and *28S*) acquired were 412 bp (*12S*), 450 bp (*16S*), 582 bp (*COII*), 594 bp (*28S*) and 1831 bp (*18S*). In order to infer the phylogenetic relationships between *Bundoksialongissimi* sp. nov. and other blattid species, we assembled a dataset with 38 samples from 33 cockroach species and two mantid species (*Bantiawerneri* and *Mantisreligiosa*) as the outgroup species downloaded from GenBank. Sequence alignment was performed through online MAFFT v.7 (Katoh et al. 2013). The Q-INS-i algorithm was used for non-coding protein genes (*12S*, *16S*, *18S*, *28S*) which were checked visually in MEGA 7 (Kumar et al. 2007); poorly aligned characters within the intergenic region were removed. The G-INS-i algorithm was selected for protein-coding genes (*COI*, *COII*) with other parameters default values, then they were manually adjusted after translation into amino acids in MEGA 7. The total length of the concatenated alignment is 4112 bp.

Using Xia’s method, implemented in DAMBE 7 (Xia 2018), the third codon position (PCG3) (I_SS_ = 0.723) was much more saturated than the first (I_SS_ = 0.294) and second codon position (I_SS_ = 0.206), indicating the third codon position is less suitable for further analyses. Due to the higher mutation saturation, the third codon was excluded in our study. Based on the combined dataset, the Maximum Likelihood (ML) and Bayesian Inference (BI) methods were used to construct the phylogenetic trees. ML inference was performed in RAxML v.7.7.1 (Stamatakis et al. 2008), using a GTRGAMMA model with 1,000 bootstrap replicates. Bayesian phylogenetic analyses was conducted in MrBayes3.2 (Ronquist et al. 2012) with the substitution models selected by PartitionFinder v.1.1.1 (Lanfear et al. 2012) as follows, GTR+I+G for *12S* and *16S*, TrNef+I+G for *18S*, TrN+I+G for *28S* and *COII*_pos12 and TIM+I+G for *COI*_pos12. Posterior distribution was estimated by Markov Chain Monte Carlo (MCMC) sampling with three hot and one cold chains and a total of 10,000,000 generations. When the average standard differentiation frequency deviation was less than 0.01, the convergence was inferred; then the first 25% of samples were discarded as the burn-in.

## ﻿Taxonomy

### ﻿ Bundoksia

Taxon classificationAnimaliaBlattodeaBlattidae

Lucañas, 2021

Bundoksia Lucañas, 2021: 1012 (Type species: Bundoksiarufocercata (Shelford, 1911), by original designation)

#### Diagnosis.

Sexual dimorphism and ocelli spots distinct. **Male.** Pronotum nearly trapezoidal or subelliptical, uneven with depressions in medium surface, posterior margin rounded. Tegmina and wings fully developed. Front femur usually type A. Tibia flattened with sparse spines. Tarsus with smooth pulvillus. Claws symmetrical and unspecialised, arolium present. The first abdominal tergum of males specialised or not. Supra-anal plate symmetrical; subgenital plate symmetrical, styli stick-like, similar size. **Male genitalia.** L2d base with several rows of serration, L2v distal part armed with spines; L3 unciform. R1 of right phallomere armed with spines. **Female.** Body thicker than the male. Pronotum parabolic, posterior margin straight. Tegmina reduced, only reaching hind margin of first abdominal tergite or metathorax; triangular or quadrate; wings reduced to small lobe. Supra-anal plate truncate, symmetrical. Subgenital plate valvular.

#### Remarks.

Lucañas (2021) mentioned that the first abdominal tergite specialised with setose gland was diagnostic for *Bundoksia* and distinguished *Bundoksia* from the other Archiblattinae by its smooth pronotum and flattened tibiae and Blattinae in terms of distinct femoral armament (meso- and metafemur sparsely armed with dissimilarly-sized spines). In previous studies, it is common that species of the same genus have or lack the abdominal tergite tergal glands, i.e. *Episymploce* (Li et al., 2020), *Scalida* (Wang et Che, 2010) in Ectobiidae and *Periplaneta* (Roth, 1994) in Blattidae. We consider that the first abdominal tergum of males, specialised or not, is not a diagnostic character of the genus *Bundoksia*, which can be distinguished from the genus, *Cartoblatta* Shelford by other characters (tegmina short and quadrate, not covering the first abdominal tergite; female supra-anal plate with hind margin cleft). Therefore, we revised the generic diagnostic ‘the first abdominal tergum of males specialised’ to ‘specialised or not’.

### ﻿Key to known species of *Bundoksia* Lucañas, 2021

**Table:** 

1	Pronotum black with one pair of yellow-orange antero-lateral markings, female tegmina quadrate	***B.rufocercata* (Shelford, 1911)**
–	Pronotum black without marking, female tegmina triangular	**2**
2	Cercus black; male: first abdominal tergite with setose gland	***B.sibuyania* Lucañas, 2021**
–	Cercus pale yellow with apex black; male: first abdominal tergite unspecialised	***B.longissima* Li & Che, sp. nov.**

### ﻿ Bundoksia longissima

Taxon classificationAnimaliaBlattodeaBlattidae

Li & Che sp. nov.

http://zoobank.org/FC409991-0647-4793-B53E-A7A39277A536

Figs 2 , 3 , 4 , 5

#### Type materials

(all deposited in SWU). ***Holotype*. China**• **Hainan**: male, Mingfenggu, Mt Jianfengling, Ledong County, 26.IV.2015, Lu Qiu & Qikun Bai leg.; SWU-B-BL0201001. ***Paratypes*. China**• **Hainan**: 9 males and 1 female, Mingfenggu, Mt Jianfengling, Ledong County, 26.IV.2015, Lu Qiu & Qikun Bai leg; SWU-B-BL0201001 to 0201010 • 1 male and 1 female, Mt Wuzhi, Wuzhishan City, 795 m alt., 18.V.2014, Shunhua Gui leg; SWU-B-BL0201101 to 0201102. **China**•**Guangxi**: 1 male, Mt Dayao, Jinxiu County, 15.VI.1974, Ping Lin & Yuliang Jia & Yaoquan Li leg; SWU-B-BL0201301 • 1 female, Mt Dayao, Jinxiu County, 7.VII.2015, Lu Qiu & Qikun Bai leg; SWU-B-BL0201201 • 1 female, Jinxiu County, 16-17.VII.2015, Lu Qiu & Qikun Bai leg; SWU-B-BL0201202. **China**•**Yunnan**: 1 male and 1 female, Mt Dawei, Pingbian County, 15-17.V.2016, Lu Oiu & Zhiwei Oiu leg; SWU-B-BL0201401, SWU-B-BL0201403 • 1 male, Jinping County, 14-16.V.2015, Jianyue Qiu leg; SWU-B-BL0201501 • 1 male, Meizi Lake, Pu’er City, 30.IV.2014, collector unknown; SWU-B-BL0201602 • 1 male, Meizi Lake, Pu’er City, 20. V. 2018, Lu Oiu & Zhiwei Oiu leg; SWU-B-BL0201601 • 1 male, Xishuangbanna Tropical Botanical Garden, Chinese Academy of Sciences, Menglun Town, Mengla County, Xishuangbanna Prefecture, 27.V.2016, Lu Oiu & Zhiwei Oiu leg; SWU-B-BL0201701 • 1 male, Wangtianshu, Mengla County, Xishuangbanna Prefecture, 24.V.2016, Lu Oiu & Zhiwei Oiu leg; SWU-B-BL0201801.

#### Other material examined

(all deposited in SWU). **China**• **Guangdong**: 1 female, Nanling National Nature Reserve, 18.VIII.2010, Haoyu Liu leg. **China**•**Guangxi**: 1 female, Mt Mao’er, Xingan County, Guilin City, 20.VIII.2020, Lu Oiu leg; 1 nymph, Mt Daming, Nanning City, 2.VII.2015, Lu Qiu & Qikun Bai leg; **China**•**Yunnan**: 3 nymphs, Mt Dawei, Pingbian County, 17.V.2016, Lu Oiu & Zhiwei Oiu leg.

#### Diagnosis.

*Bundoksialongissima* sp. nov., differs from the two known species, *B.rufocercata* (Shelford, 1911) and *B.sibuyania* Lucañas, 2021 by the following characteristics: 1) pronotum: with slightly thickened lateral margin; 2) mid- and hind- femur with only distal spines on ventral margin; 3) the first abdominal tergite unspecialised. In addition, *Bundoksialongissima* sp. nov. can be distinguished from *B.rufocercata* as follows: pronotum black and female tegmina triangular in the former, whereas pronotum with yellow orange marking and female tegmina quadrate in *B.rufocercata*.

#### Measurements

**(mm). Male.** Body length including tegmen: 22.6–26.4; body length: 17.0–19.4; pronotum length × width: 3.8–5.0 × 5.0–5.9; interantennal distance: 1.25–1.39; interocular distance: 0.81–1.08; head length × width: 2.95–3.35 × 2.91–3.22; tegmina length: 18.9–23.4; approximate length ratio of 3^rd^–5^th^ segments of maxillary palps about 1:0.75:1. **Female.** Body length: 18.8–21.6; pronotum length × width: 4.5–5.0 × 6.6–7.4; interantennal distance: 1.62–1.83; interocular distance: 1.55–1.84; head length × width: 3.84–4.49 × 0.80-1.05; tegmina length: 4.42–5.74; approximate length ratio of 3^rd^–5^th^ segments of maxillary palps about 1:0.75:1.

#### Description.

**Figure 2.**
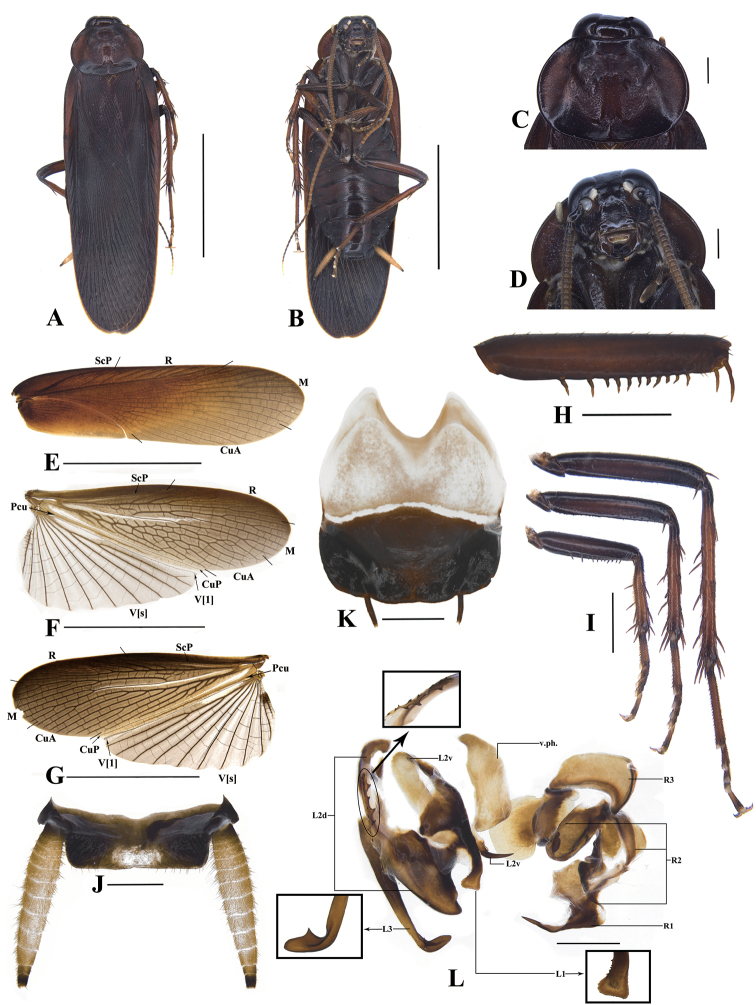
*Bundoksialongissima* Li & Che, sp. nov. (male) **A** dorsal view **B** ventral view **C** pronotum **D** head **E** tegmen **F** right hind wing **G** left hind wing **H** front femur **I** leg (front, mid, hind) **J** supra-anal plate **K** subgenital plate **L** phallomere; Scale bars: 10.0 mm (**A, B, E–G**); 2.0 mm (**I**); 1.0 mm (**H, J–L**).

**Male. Colouration.** Body unicoloured dark reddish-brown to blackish-brown, except the following portions: ocelli white; clypeus light brown or yellowish-brown; antennae yellowish-brown, basal and distal portion darker, apex distinctly light coloured; wings with anal area transparent, the remaining part yellowish-brown; tibiae and tarsi slightly light coloured (reddish-brown), except the joints; cerci yellowish, apex segment black, with white tip (Fig. 2A–B).

Body slender, flattened. ***Head*.** Vertex unconcealed by pronotum, smooth, slightly punctured. Interocular space wide, as wide as the distance between ocelli, narrower than the distance between antennal sockets. Ocelli oval (Fig. 2D). **Thorax.** Pronotum nearly subelliptical, wider than long. Surface smooth, disc with unequal-sized punctures. The border of pronotum thickening, anterior margin slightly elevated, lateral margins rounded, hind margin slightly arched (Fig. 2C). **Tegmina and wings.** Tegmina fully developed, extending well beyond the end of abdomen. Outer margins of tegmina straight, apex of tegmen rounded. Tegmen with ScP slightly curved; R ended at the margin about 1/3 from the apex; M and Cu with numerous branches (Fig. 2E). Wings with ScP slightly vague; M with a dichotomy in base, pseudostem distinct; CuA simple and linear or lattice-like; CuP simple and obvious (Fig. 2F, G). **Legs.** Front femur type A2 (ending with a large, curved spine and a smaller spine, hind margin of front femur with a row of rough, distant spaced spins) (Fig. 2H); tibia flattened with sparse spines; tarsus with large tarsal pulvillus. Mid- and hind femur with only distal spines on ventral margin. Hind metatarsus obviously longer than the remaining tarsomeres combined (Fig. 2I). Claws symmetrical and unspecialised, arolium large. **Abdomen.** Supra-anal plate symmetrical, quadrate, with hind angles rounded, hind margin straight, median less sclerotised. Paraprocts similar, hind margin straight, central areas sclerotised. Cerci distinct pubescent ventrally, smooth dorsally, apex truncated, with membrane (Fig. 2J). Subgenital plate nearly symmetrical, styli similar, distant (Fig. 2K).

***Male genitalia*.** Left phallomere complex, distal part of L1 enlarged, edge with dense minute sawtooth; L2d base part with two or three rows of serrations, L2v distal part with spines; L3 unciform and apex blunt or slightly acuminate, curved part has an inward spinous protuberance. R1 of right phallomere with one or two spines with the sizes of the two spines varied; R2 expanded, irregular; R3 broad and slightly curved, likely spoon-shaped (Fig. 2L).

**Figure 3.**
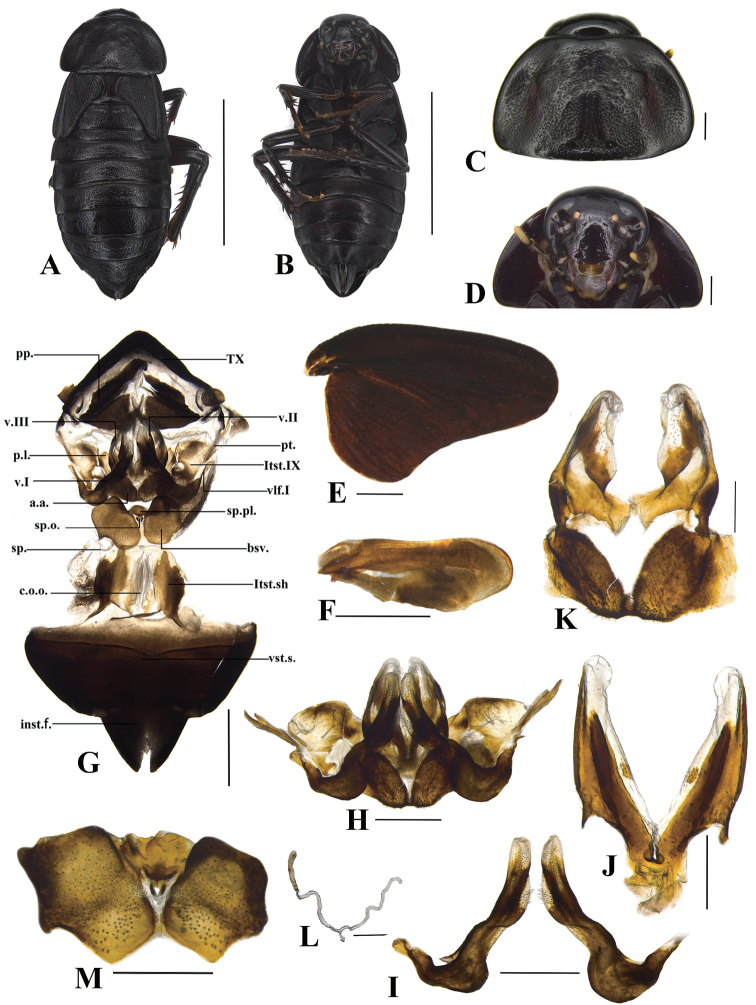
*Bundoksialongissima* Li & Che, sp. nov. (female) **A** dorsal view **B** ventral view **C** pronotum **D** head **E** tegmen **F** hind wing **G** genitalia, posterior view **H** valves and accessory sclerites **I** first valvule (v.I.) **J** second valvule (v.II.) **K** third valvule (v.III) and anterior arch **L** spermatheca (sp.) **M** basivalvula (bsv.); Scale bars: 10.0 mm (**A, B**); 1.0 mm (**C–I, M**); 0.5mm (**K, J, L**).

**Female** (Fig. 3A–M)**. Description. Colouration.** Body darker than male. (Fig. 3A, B).

Body thicker than the male. ***Head*.** Interocular space wider than the distance between ocelli, narrower than the distance between antennal sockets (Fig. 3D). **Thorax.** Pronotum nearly trapezoidal, punctuated, hind angles rounded, posterior margin almost straight (Fig. 3C). **Tegmina and wings.** Tegmina reduced, only reaching hind margin of first abdominal tergite; triangular, thickened, angles rounded (Fig. 3E); wings small lobed (Fig. 3F). **Legs.** Femur and tibia stronger than male. **Abdomen.** Hind margin of tergum X (TX) blunt. Paraprocts (pp.) wide and symmetrical, with the gap between pp. narrow. Subgenital plate divided at the end, the middle with distinct intersternal fold (inst.f.) (Fig. 3G). **Genitalia.** The base of first valve (v.I.) (Fig. 3I) more sclerotised and fused with first valvifer (vlf.I), vlf.I short. Laterosternite IX (ltst.IX) large and sheet-like, with outer margin hyaline, fused with paratergites (pt.). Second valve (v.II) small, slender, the base fused, connecting to third valve (v.III) by membrane (Fig. 3J). Posterior lobes of valvifer II (p.l.) sclerotised, cricoid, distal uneven and fused with ltst.IX. Third valve (v.III) large, the base sclerite convex, highly sclerotised (Fig. 3K). Anterior arch (a.a.) hip-shaped, the base deeply concave, with dense spines. Spermathecal plate (sp.pl.) slightly sclerotised, fused with basivalvula (bsv.). Spermathecal opening (sp.o.) located at the base of sp.pl., with small sclerites on two sides and highly sclerotised. Spermatheca (sp.) with two branches near the base and one branch with a rod-shaped enlargement distally (Fig. 3L). Basivalvulae (bsv.) developed and divided into two parts, with bristle-shaped spins (Fig. 3M). Laterosternal shelf (ltst.sh.) developed and symmetrical, extending backwards. Vestibular sclerite (vst.s.) unclear in outline; the base with a transverse sclerotised plate.

**Figure 4.**
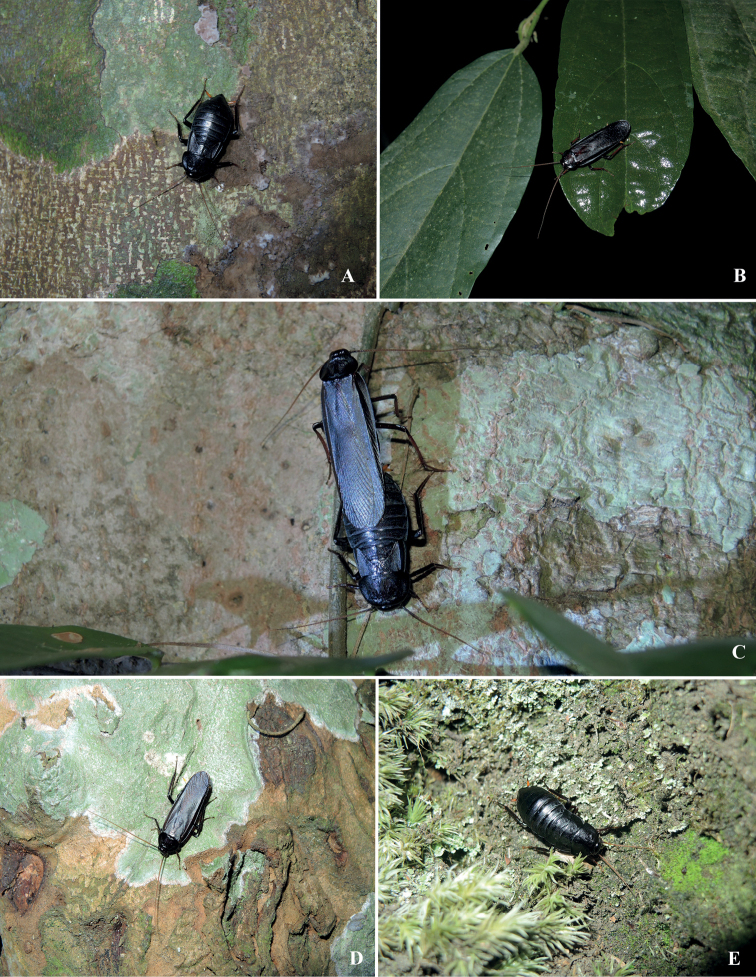
Habitats of *Bundoksialongissima* Li & Che, sp. nov. **A** female on tree trunk **B** male on tree leaf **C** mating on tree trunk (**A–C** from Jianfengling, Ledong, Hainan) **D** male on tree trunk (Xishuangbanna Tropical Botanical Garden, Yunnan) **E** nymph on the moss-covered ground (Daweishan, Pingbian, Yunnan). Photographed by Lu Qiu.

**Nymph.** Wingless, with light body colour and thin body size, compared to females. Other characteristics are similar to females (Fig. 4E).

#### Etymology.

The scientific epithet is derived from the Latin word *longissimus*, referring to the long and narrow body.

#### Ecology.

According to our collecting information, *Bundoksialongissima* is active at night to forage and mate. It is distributed mainly on tree trunks, a few on leaves (Fig. 4). Once frightened, the female will emit an acidic liquid (lemon smell), whose specific components have not been analysed.

#### Remarks.

**Figure 5.**
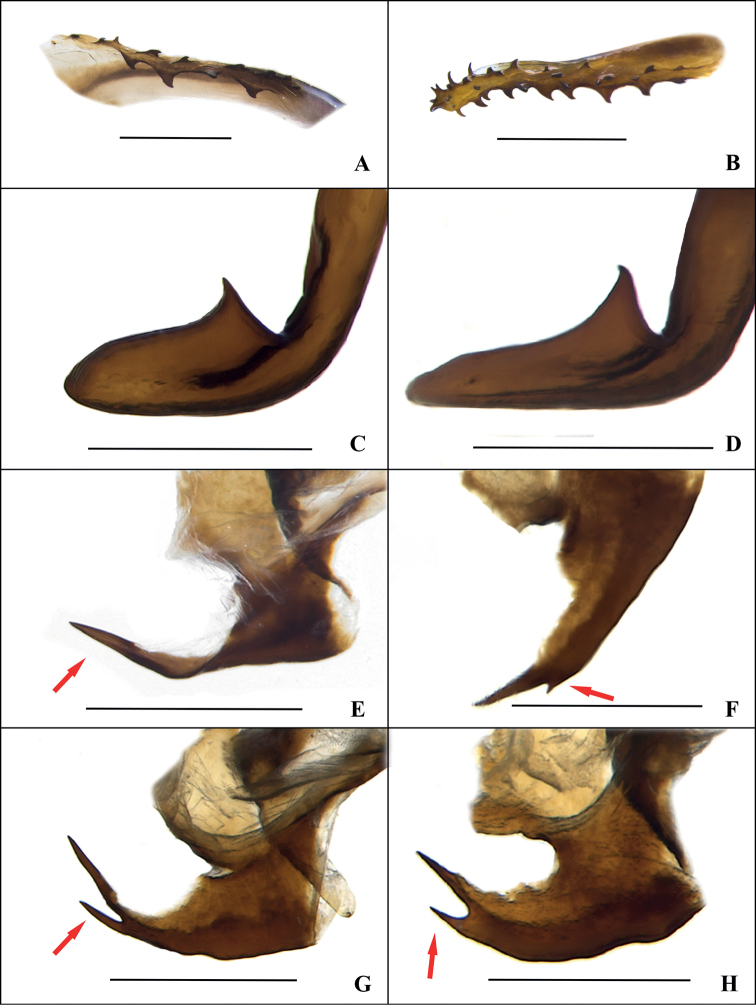
Variations on the male genitalia of *Bundoksialongissima* Li & Che, sp. nov. **A–B** L2d of left phallomere (**A** Hainan **B** Yunnan) **C–D** L3 of left phallomere (**C** Hainan **D** Yunnan) **E–H** R1 of right phallomere (**E** Hainan **F–H** Yunnan). Scale bars: 0.5 mm (**A–D**); 1.0 mm (**E–H**).

Samples from Yunnan show a range of slight morphological differences (mainly male genitalia) compared with Hainan and Guangxi: 1) the samples from Hainan and Guangxi with L2d base part with two-rows of serration (Fig. 5A), but the one from Yunnan with L2d base part with three-rows of serration (Fig. 5B); 2) the samples from Hainan and Guangxi with L3 unciform and apex blunt (Fig. 5C), but the Yunnan specimens with L3 unciform and apex slightly acuminate (Fig. 5D); 3) the samples from Hainan and Guangxi with R1 apically unforked (Fig. 5E), but three samples from Yunnan with R1 apically forked (Fig. 5 F–H). On the other hand, only the right hind-wing of the holotype was found as CuA with lattice-like, angular cross-veins, while the left hind-wing of the holotype and hind-wings of the remaining samples were simple and linear. In addition, all female individuals of *Bundoksialongissima* sp. nov. appear to be highly conserved in terms of external morphology and genital structure (Fig. 3). Given this, it is difficult to distinguish them, based only on these slight variations in the shape of the male genitalia, so we temporarily consider them to be intraspecific variations in morphology.

Due to the similarities in the femoral armature, *Catarahainanica* described by Liu et al. (2017) might belong to *Bundoksia* or perhaps a closely-related genus. However, Liu et al. (2017) only described a single female nymph, so male adults of *C.hainanica* should be carefully examined to confirm the above hypothesis in the future.

#### Known distribution.

China (Hainan, Guangxi, Yunnan, Guangdong Province)

## ﻿Results

### ﻿Relationships amongst different populations of *Bundoksialongissima* sp. nov., based on COI data

Pairwise genetic distances in *Bundoksialongissima* sp. nov. range from 0.0 to 7.2%, with an average of 3.35% (Suppl. material 3: Table S3). The largest distance 7.2% exists between Xishuangbanna Botanical Garden, Yunnan (YNBG1) and Jianfengling of Hainan (HIJFL1, HIJFL2, HIJFL7), Mt. Daming of Guangxi (GXDMM1). Combined with male and female morphological characteristics, including and genital structures (Fig. 2, 3), despite the large genetic distance and other existing slight variations, all samples studied here are still treated as one species, *Bundoksialongissima* sp. nov.

**Figure 6.**
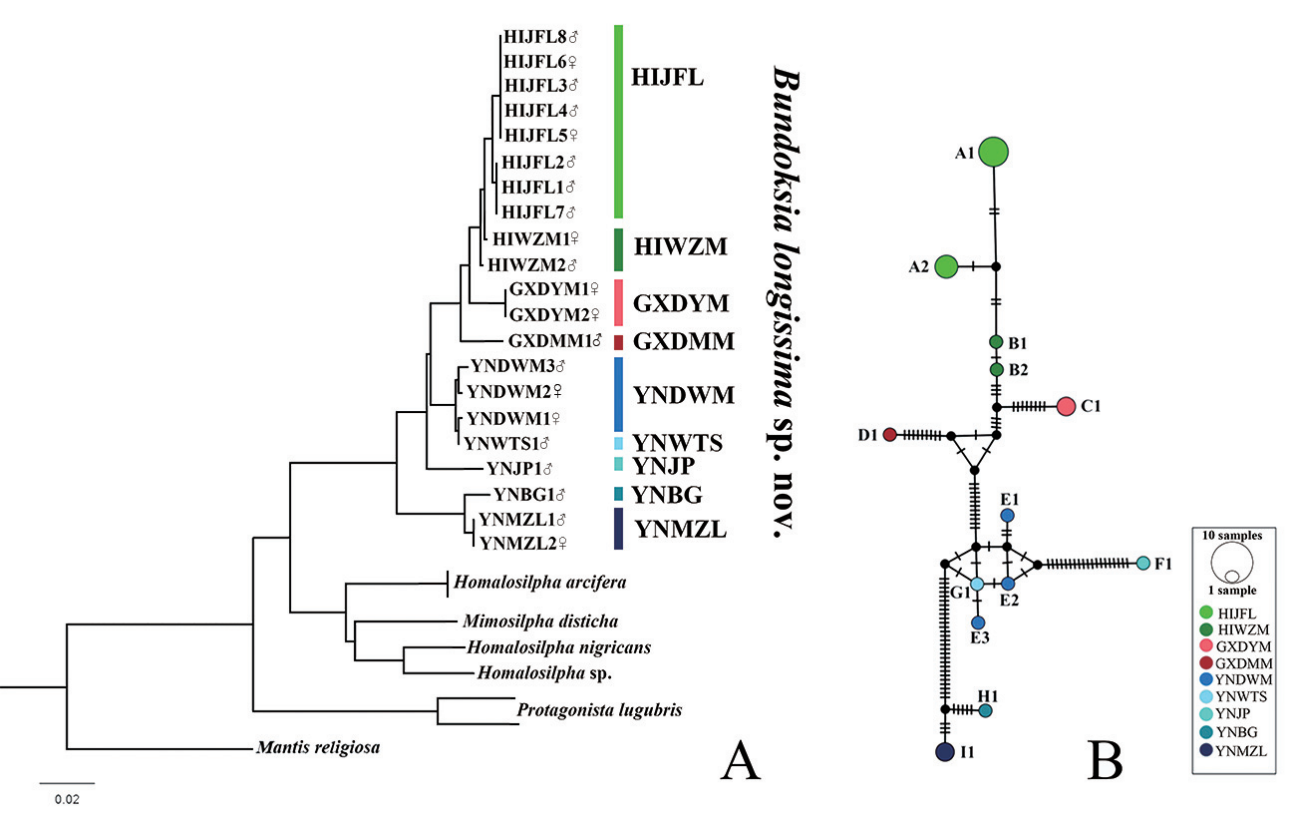
Neighbour-Joining (NJ) tree and haplotype network structure, based on COI data of *Bundoksialongissima* sp. nov. **A**NJ tree **B** haplotype network **A–B** different colours represent different populations and the black circles represent missing haplotypes in the mutation process. The colour of all circles of the NJ tree and haplotype network is consistent. More details of abbreviation of locations are included in Suppl. material 1: Table S1.

In the NJ tree, all individuals of *Bundoksialongissima* sp. nov. are clustered together to form a monophyletic group (Fig. 6A), solving the sexual dimorphism of *Bundoksialongissima* sp. nov. Samples from Guangxi are more related to those from Hainan than the others. Yunnan samples are split into several distinct groups in the NJ tree, which corresponds with the types of variations on R1. These groups represent different geographical locations from Yunnan Province.

Thirteen haplotypes were recorded from 21 *COI* sequences of *Bundoksialongissima* sp. nov. (Fig. 6B), of which, four haplotypes (A1, A2, B1, B2) were from Hainan, two (C1, D1) from Guangxi and seven (E1, E2, E3, F1, G1, H1, I1) from Yunnan. The haplotype network showed that there were no shared haplotypes amongst different geographic populations. Haplotypes from Yunnan except E1, E2, E3 and G1, were connected via at least 18 mutational steps. Haplotypes from Hainan were well connected via a maximum of five mutations.

### ﻿Taxonomic affinities of *Bundoksia* inferred from two phylogenetic analyses

**Figure 7.**
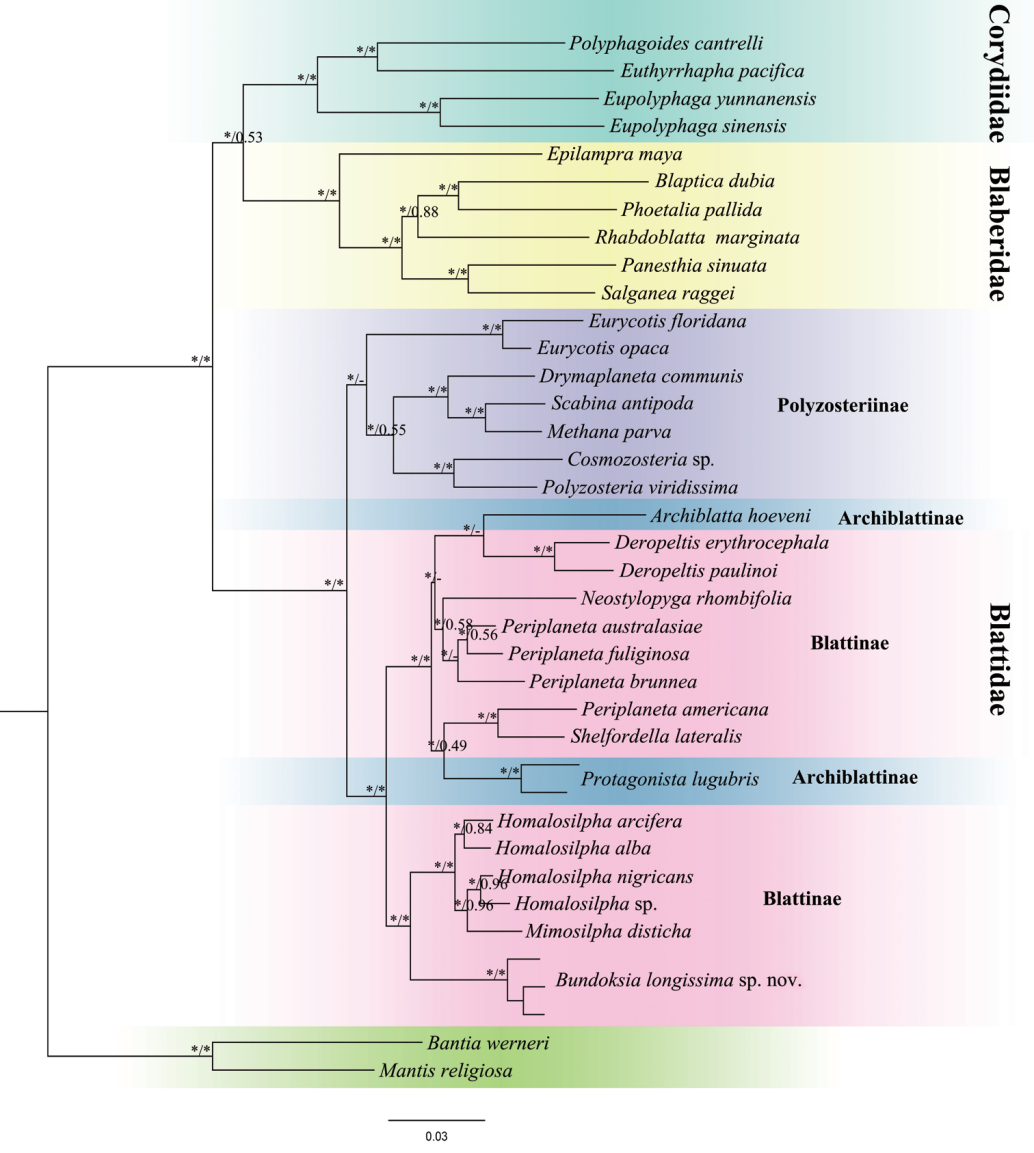
Maximum Likelihood (ML) tree of cockroaches inferred from four mitochondrial markers *12S* rRNA, *16S* rRNA, *COI, COII* and two nuclear markers *28S* rRNA, *18S* rRNA. Branch support labels are as follows: bootstrap supports of the Maximum-Likelihood tree/Bayesian posterior probabilities of the Bayesian tree; (*) indicate the branch label of given analysis is maximal (i.e. MLB = 100 or BPP = 1.0), (-) means the node is absent for the given analysis.

Our Maximum Likelihood and Bayesian Inference analyses yielded almost identical topologies, based on the concatenated dataset (Fig. 7, Suppl. material 4: Fig. S1). In our study, Blattidae was recovered to be monophyletic with high support values (MLB = 100, BPP = 1) (Fig. 7). Two major lineages of Blattidae, Polyzosteriinae and Archiblattinae + Blattinae, were revealed in both analyses. According to our inferred trees, both Archiblattinae and Blattinae were paraphyletic. *Bundoksialongissima* sp. nov. was found to be the sister group of *Homalosilpha* + *Mimosilpha* with high support (MLB = 100, BPP = 1). In addition, *Bundoksialongissima* sp. nov., together with other Blattinae and Archiblattinae members, form a monophyletic group with strong support, as the sister group of Polyzosteriinae.

## ﻿Discussion

The haplotype network diagram (Fig. 6B) showed that there was no shared haplotype amongst geographical populations and suggested that the Hainan haplotypes are relatively less diverse, while the haplotypes from Yunnan showed more genetic diversification. In addition, the pairwise genetic distances amongst samples in Yunnan varied greatly from 0 to 7.2%. The NJ tree showed the same result, especially samples from Yunnan forming several distinct groups. We suggest that the larger genetic distance and morphological differences might be related to the natural barriers (mountains or rivers in Yunnan), which reduce gene communication amongst populations, which might lead to a high genetic diversity.

*Mimosilpha* and *Homalosilpha* are closely related to *Bundoksialongissima* sp. nov. according to our phylogenetic reconstruction, which could be distinguished from *B.longissima* sp. nov. by the flattened tibiae, the distinct femoral armament and the maculae bearing in the pronotum. In previous works, the subfamilies Archiblattinae and Blattinae were recovered as monophyletic (Inward et al. 2007; Djernaes et al. 2015; Legendre et al. 2015) or paraphyletic (Liao et al. 2021). According to our inferred trees, Archiblattinae and Blattinae are paraphyletic. Archiblattinae is embedded in Blattinae, indicating Archiblattinae might be a synonym of Blattinae. Lucañas (2021) also believed that the genus *Bundoksia* not only possesses the characteristics of the subfamily Archiblattinae (the distinct femoral armament, meso- and metafemur sparsely armed with dissimilarly-sized spines), but also those of Blattinae (the smooth pronotum and flattened tibiae). Wang et al. (2016) discovered that the male genitalia of Archiblattinae was similar to that of Blattinae. Therefore, in follow-up works, we need more molecular data and morphological evidence to solve the relationship between Archiblattinae and Blattinae and then settle the subfamilial status of the genus *Bundoksia*.

## Supplementary Material

XML Treatment for Bundoksia

XML Treatment for Bundoksia longissima

## Supplementary materials

Supplementary material 1 Table S1 Data type xlsx file Explanation note Samples used in Neighbor joining (NJ) tree and haplotype network: GenBank accession numbers, number of location, sample ID, sample localities and data and voucher numbers. https://binary.pensoft.net/file/643520This dataset is made available under the Open Database License (http://opendatacommons.org/licenses/odbl/1.0/). The Open Database License (ODbL) is a license agreement intended to allow users to freely share, modify, and use this Dataset while maintaining this same freedom for others, provided that the original source and author(s) are credited.Yong Li, Xinxing Luo, Jiawei Zhang, Zongqing Wang, Yanli Che

Supplementary material 2 Table S2 Data type xlsx file Explanation note Specimen used in molecular phylogenetic analysis (ML and BI), with details of family, sample id, collecting localities, references and GenBank accession numbers. https://binary.pensoft.net/file/643521This dataset is made available under the Open Database License (http://opendatacommons.org/licenses/odbl/1.0/). The Open Database License (ODbL) is a license agreement intended to allow users to freely share, modify, and use this Dataset while maintaining this same freedom for others, provided that the original source and author(s) are credited.Yong Li, Xinxing Luo, Jiawei Zhang, Zongqing Wang, Yanli Che

Supplementary material 3 Table S3 Data type xlsx file Explanation note Pairwise genetic distances with standard errors of Bundoksialongissima sp. nov. calculated by using K2P model using cytochrome oxidase subunit I (COI) gene sequences in MEGA. Bold text denotes the standard errors of distances and black denotes pairwise genetic distances. https://binary.pensoft.net/file/643522This dataset is made available under the Open Database License (http://opendatacommons.org/licenses/odbl/1.0/). The Open Database License (ODbL) is a license agreement intended to allow users to freely share, modify, and use this Dataset while maintaining this same freedom for others, provided that the original source and author(s) are credited.Yong Li, Xinxing Luo, Jiawei Zhang, Zongqing Wang, Yanli Che

Supplementary material 4 Figure S1 Data type jpg file https://binary.pensoft.net/file/643526This dataset is made available under the Open Database License (http://opendatacommons.org/licenses/odbl/1.0/). The Open Database License (ODbL) is a license agreement intended to allow users to freely share, modify, and use this Dataset while maintaining this same freedom for others, provided that the original source and author(s) are credited.Yong Li, Xinxing Luo, Jiawei Zhang, Zongqing Wang, Yanli Che
